# Structural Basis of Ultralow Capacitances at Metal–Nonaqueous Solution Interfaces

**DOI:** 10.1021/jacs.4c12443

**Published:** 2025-01-27

**Authors:** Juan Chen, Zengming Zhang, Xiaoting Yin, Chenkun Li, Fengjiao Yu, Yuping Wu, Jiawei Yan, Jun Huang, Yuhui Chen

**Affiliations:** 1 State Key Laboratory of Materials-Oriented Chemical Engineering, College of Chemical Engineering, Nanjing Tech University, Nanjing 211816, China; 2 Institute of Energy Technologies, IET-3: Theory and Computation of Energy Materials, Forschungszentrum Jülich GmbH, Jülich 52425, Germany; 3 State Key Laboratory of Physical Chemistry of Solid Surfaces and College of Chemistry and Chemical Engineering, Xiamen University, Xiamen 361005, China; 4 Key Laboratory of Energy Thermal Conversion and Control of Ministry of Education, School of Energy and Environment, Southeast University, Nanjing 210096, P. R. China; 5 Theory of Electrocatalytic Interfaces, Faculty of Georesources and Materials Engineering, RWTH Aachen University, Aachen 52062, Germany

## Abstract

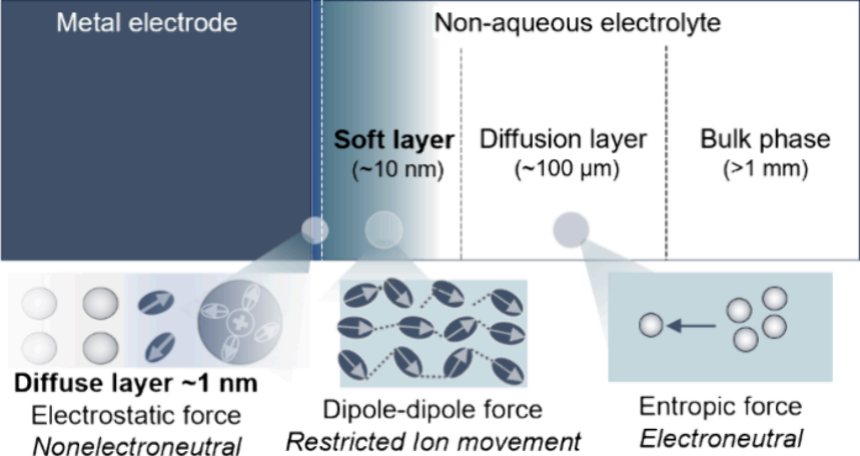

Metal–nonaqueous solution interfaces, a key to many electrochemical technologies, including lithium metal batteries, are much less understood than their aqueous counterparts. Herein, on several metal–nonaqueous solution interfaces, we observe capacitances that are 2 orders of magnitude lower than the usual double-layer capacitance. Combining electrochemical impedance spectroscopy, atomic force microscopy, and physical modeling, we ascribe the ultralow capacitance to an interfacial layer of 10–100 nm above the metal surface. This nanometric layer has a Young’s modulus around 2 MPa, which is much softer than typical solid-electrolyte interphase films. In addition, its AC ionic conductivity is 4-to-5 orders of magnitude lower than that of the bulk electrolyte. The temperature dependencies of the AC ionic conductivity and thickness suggest that the soft layer is formed from metal-mediated, dipole–dipole interactions of the nonaqueous solvent molecules. The observed soft layer opens new avenues of modulating battery performance via rational design of ion transport, (de)solvation, and charge transfer in this interfacial region.

## Introduction

The interfacial region between the solid electrode and electrolyte solution, broadly termed the electrical double layer (EDL), is a central topic in electrochemistry because charge transfer reactions occur in this highly heterogeneous region.^1−9^ Most research on the EDL focuses on metal–aqueous solution interfaces, while much less attention has been given to metal–nonaqueous solution interfaces. Recent decades have witnessed the rapid development of lithium-ion batteries, warranting a level of understanding of the EDL at metal–nonaqueous solution interfaces commensurate with its technological importance.^6−8,10−16^

The interfaces in nonaqueous lithium-ion batteries are insufficiently understood, in a large part, because the nonaqueous electrolyte often decomposes spontaneously at the electrode surface, forming a solid-electrolyte interphase (SEI) layer.^17−19^ A good SEI layer is ionically conductive while electronically insulating, impeding further electrolyte decomposition at the electrode surface. The crucial SEI introduces at least two EDLs, namely, one at the inner solid–solid interface and the other at the outer solid–liquid interface.^20−23^ While it is certainly important to study these two EDLs after the formation of the SEI, we hold the view that a fundamental understanding of the EDL before the formation of the SEI is even more important. This is because the pristine EDL determines the crucial local reaction conditions, namely, the local densities of cations, anions, and solvent molecules, for forming the SEI. Very recently, Wu et al.^11^ developed a joint molecular dynamics and density functional theory method to study the pristine EDL at an atomic level. This study unravels molecular insights into how the EDL regulates the formation of the SEI. Similar computational insights are being pursued in other types of batteries, including but not limited to the alkaline-metal batteries and the magnesium metal batteries.^24^

Currently, knowledge obtained for the EDL at metal–aqueous solution interfaces constitutes the basis for describing its counterpart in nonaqueous solutions.^25−27^ This knowledge transfer is credible only when an adequate understanding of the difference between the interface in aqueous electrolytes and that in nonaqueous electrolytes is obtained priori. As regards the EDL at metal–aqueous solution interfaces, the classical Gouy–Chapman–Stern–Grahame (GCSG) model describes it as a serial connection of an inner Helmholtz layer for specifically adsorbing ions, an outer Helmholtz layer for nonspecifically adsorbing ions, and a diffuse layer stretching toward the bulk solution.^2,4,28,29^ As a hallmark of the GCSG model, the differential double layer capacitance (*C*_dl_) profile resembles a camel shape with the minimal capacitance obtained at the potential of zero charge (PZC). As regards a normal concentration of 1 M, the minimal *C*_dl_ is on the order of tens of μF/cm^2^.^30,31^

Herein, we report on an interfacial layer with an ultralow capacitance, 2 orders of magnitude smaller than the normal *C*_dl_, for a variety of metal–nonaqueous solution interfaces. This ultralow capacitance is at odds with the traditional GCSG model, calling for an overhaul of the current understanding of metal–nonaqueous solution interfaces. Our combined electrochemical and mechanical measurements show that this interfacial layer possesses distinct properties compared to the EDL, typical SEIs, and the bulk solution. It is ionically conductive, but its AC conductivity is 5 orders of magnitude lower than the bulk values. It is quasi-solid but much softer than typical SEIs in Li-ion batteries. A wide range of factors influencing the ultralow capacitance, including the metal nature, the solvent nature, and the temperature, are examined, which, taken together, depicts a new model for the EDL at metal–nonaqueous solution interfaces.

## Results and Discussion

### Anomalous High-Frequency Semicircles

**Figure 1:**
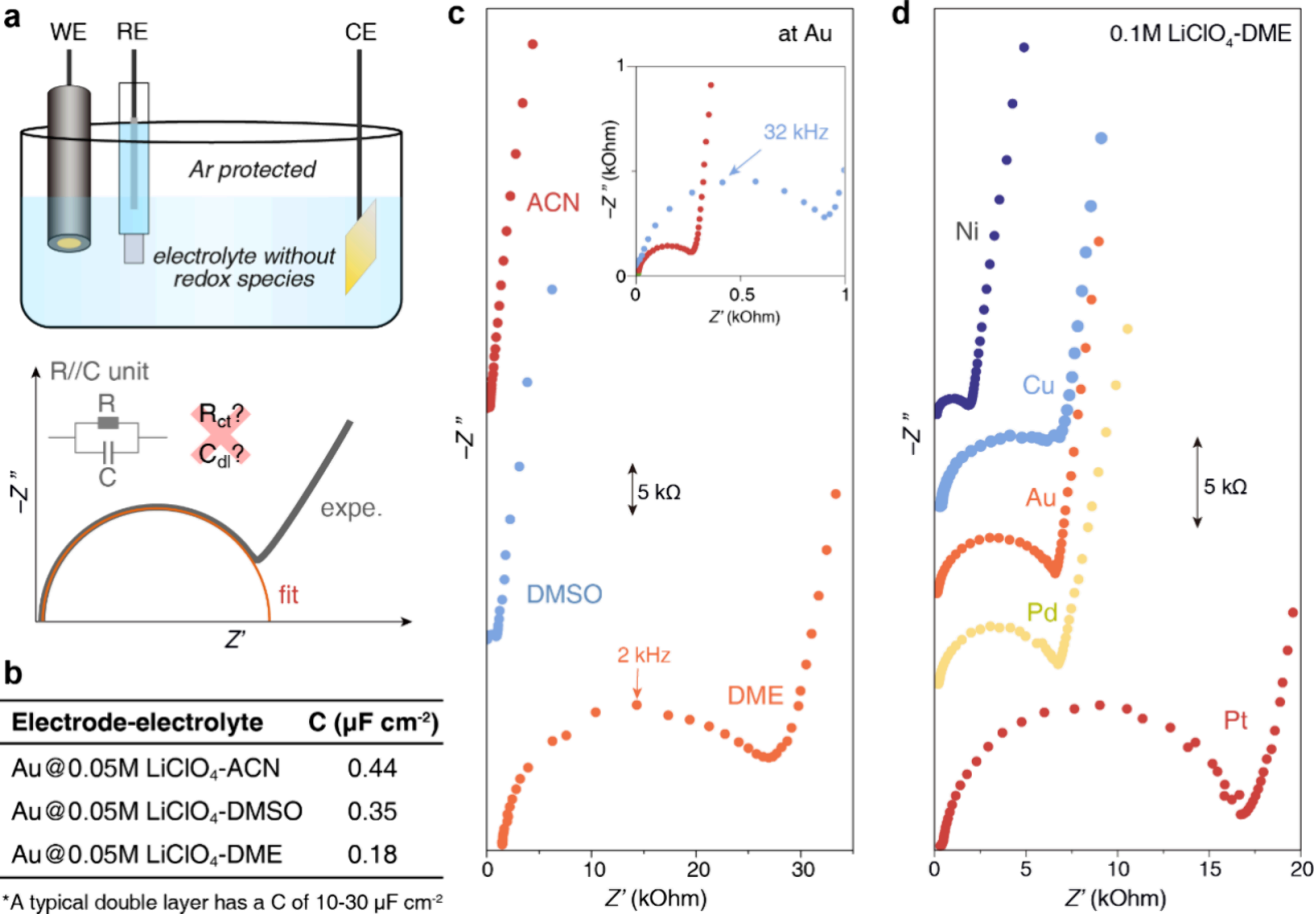
Ultralow high-frequency capacitance at the metal–nonaqueous solution interfaces. (a) Schematics of the experimental setup and an equivalent circuit model for the high-frequency semicircle. As rationalized in the main text, the resistance *R* is not related to charge transfer, and the capacitance *C* is not the differential EDL capacitance; (b) ultralow capacitance estimated from the high-frequency semicircle in the Nyquist plots. (c, d) Nyquist plots of the EIS (c) in various solvents at a Au electrode and (d) at various working electrodes in 0.1 M LiClO_4_-DME at room temperature. The plots were shifted vertically for clarity, and the scale bars of the *y*-axis are 5 kΩ in (c,d).

We first employ the nondestructive, operando electrochemical impedance spectroscopy (EIS) to investigate the electrochemical interfaces in nonaqueous systems without apparent redox reactions. EIS measurements involve applying a sufficiently small voltage perturbation, usually not larger than 20 mV, to an electrochemical cell under stationary conditions and measuring the current response. After Fourier transformation of the voltage stimulus and current response, the impedance is obtained as the ratio of voltage to current in the frequency domain. The complex impedance is usually displayed in Nyquist plots with the real part of the impedance on the horizontal axis and the negative imaginary part on the vertical axis.^32,33^ The details of experiments are described in Materials and Methods. Briefly, a three-electrode glass cell was assembled, and EIS was collected at the PZC inside an Ar-filled glovebox, as shown in Figure 1a. A well-cleaned gold (Au) electrode, a AgCl/Ag electrode behind a glass frit, and a platinum (Pt) wire served as the working, reference, and counter electrode, respectively. Three electrolytes, i.e., acetonitrile (ACN), dimethyl sulfoxide (DMSO), and 1,2-dimethoxyethane (DME), containing 0.05 M lithium perchlorate (LiClO_4_) were examined. Nyquist plots of EIS show a distinct semicircle in all three electrolytes although their sizes are different; see Figure 1c. This high-frequency semicircle is observed not only at the Au surface in 0.1 M LiClO_4_-DME but also at different working electrodes, i.e., nickel (Ni), Pt, palladium (Pd), and copper (Cu) (Figure 1d).

To exclude the possibility of a charge transfer process, EIS measurements are conducted at various electrode potentials. If the high-frequency semicircle is assigned to a charge transfer process, the size of the semicircle, equivalent to the charge transfer resistance, would decrease at increasing overpotentials. However, as shown in Figure S1a, the Nyquist plots at ±100 and ±200 mV vs the PZC almost overlap with the plot at the PZC, which strongly suggests that this semicircle is not associated with any interfacial charge transfer process.

To exclude the influence of undesired side reactions of the electrolyte impurities, the LiClO_4_ salt has been recrystallized to remove the impurities, and the electrolyte solvents have been distilled under vacuum or Ar. After distillation, the solvents were moved and stored in a glovebox. The electrolytes were prepared in the glovebox and the water concentration in electrolytes was below 4 ppm. If the semicircle in the LiClO_4_-DME electrolyte originates from the impurities in LiClO_4_, then the Nyquist plot in the LiClO_4_-H_2_O electrolyte should show a similar semicircle. However, as shown in Figure S2, the high-frequency semicircle disappears in a 0.1 M LiClO_4_-H_2_O electrolyte for both Au and Pt electrodes. The ^1^H NMR of the electrolyte in D_2_O does not show any signals from impurities (Figure S3). Additionally, EIS measurements were conducted in an Ar-filled glovebox to prevent possible O_2_-involved reactions in nonaqueous electrolytes, such as oxygen reduction reactions.

To rule out the possibility of a passivation layer at the surface of the Au electrode, the Au electrode has been well polished and electrochemically cleaned in perchloric acid solution using cyclic voltammetry according to a standard protocol prior to transferring into an Ar-filled glovebox.^34^ The measured EIS still shows a semicircle in the high-frequency region. We also tried to polish the Au electrode with an extra fine sandpaper (10,000 mesh) inside a glovebox to obtain a fresh surface without passivation, but it did not alter the results much.

If the passivation layer at the Au surface came from the side reactions of electrolytes, *e.g.,* the electrolyte decomposition at the lithium surface, the passivation layer thickness and the semicircle size are expected to increase with the immersion time. However, Figure S1b shows that the high-frequency semicircle does not grow with the immersion time. Interestingly, when we quickly transferred this Au electrode from the LiClO_4_-DME solution into the LiClO_4_-H_2_O solution, the high-frequency semicircle immediately disappeared (Figure.S4).

As an intermediate summary, EIS measurements have revealed a high-frequency semicircle at metal–nonaqueous solution interfaces. Systematic control experiments reveal that the high-frequency semicircle is not associated with a charge transfer reaction or a passivation thin film on the metal surface. Moreover, it instantly disappears when the nonaqueous solution is replaced with an aqueous solution, unambiguously indicating that this semicircle is a unique behavior of metal–nonaqueous solution interfaces.

### Ultralow Interfacial Capacitance and Its Origin

We quantify the high-frequency semicircle parameters by using a physics-based impedance model for ideally polarizable interfaces. The model describes ion transport driven by electrostatic potential and concentration gradients within an electrolytic layer of thickness *L* and ionic conductivity σ. At the PZC, the EIS is analytically obtained as,^35^
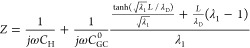
1where  is the Helmholtz capacitance with ϵ_HP_ and δ_HP_ being the dielectric constant and thickness of the space between the metal surface and the Helmholtz plane, respectively,  is the Gouy–Chapman capacitance with ϵ being the dielectric constant of the electrolyte solution and  the Debye length, and  is a frequency-dependent unitless variable with *D* being the ionic diffusion coefficient. In the high-frequency region, eq 1 is reduced to
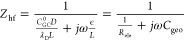
2where  is the ionic resistance and  is the geometric capacitance of this layer. We should distinguish *C*_geo_ from the EDL capacitance . *C*_geo_ is obtained in the high-frequency range, where the EDL charging/discharging does not occur.

If we denote the resistance and capacitance of the high-frequency semicircle with *R*_hf_ and *C*_hf_, respectively, the physical model allows us to determine the thickness and ionic conductivity of the electrolytic layer as follows:
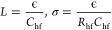
3

Equation 3 indicates that, provided with ϵ, we can determine *L* and σ from the EIS measurements. We use the bulk permittivity of the electrolyte solution for ϵ since *L* is found to be on the order of tens of nanometers and the permittivity does not change significantly on this scale.^36^ Instead, the reported permittivity decrement occurs in the subnanometric region in the EDL.^37^ The temperature dependence of the permittivity is considered in the analysis of the temperature-varying EIS results. Specifically, the relationship determined previously from molecular dynamics simulations is adopted here.^37^ For mixed solvents, we use a linear relation to estimate the permittivity. Detailed expressions are provided in the Supporting Information.

This interfacial layer is estimated to be several tens of nanometers in a 100 mM LiClO_4_ electrolyte, much thicker than expected. In contrast, an EDL is generally ∼1 nm-thick in the same electrolyte.^30,38^ To verify the thickness of this interfacial layer, we carried out the same EIS experiments on a polished planar Au electrode and a roughened Au electrode. The Au electrode was roughened by following an electrochemical procedure used for surface-enhanced Raman spectrometry (see the Supporting Information). The roughened electrode was rinsed with water to maintain a clean surface without ion residuals. The SEM images in Figure S5 show bumps of around 10 nm on the metal surface, consistent with the literature.^39^ After roughening, the surface area of the electrode greatly increases (Figure S6a). If the thickness of this interfacial layer was as thin as an EDL, its capacitance would increase by an order of magnitude after roughening simply due to the larger surface area. However, the Nyquist plot and estimated *C*_geo_ of the roughened Au electrode are very similar to those of a polished Au electrode (Figure S6b). Therefore, the thickness of this interfacial layer is at least on the same order of magnitude as the size of the bumps, namely, around 10 nm. The results further confirm that *C*_geo_ is not attributed to the EDL.

**Figure 2:**
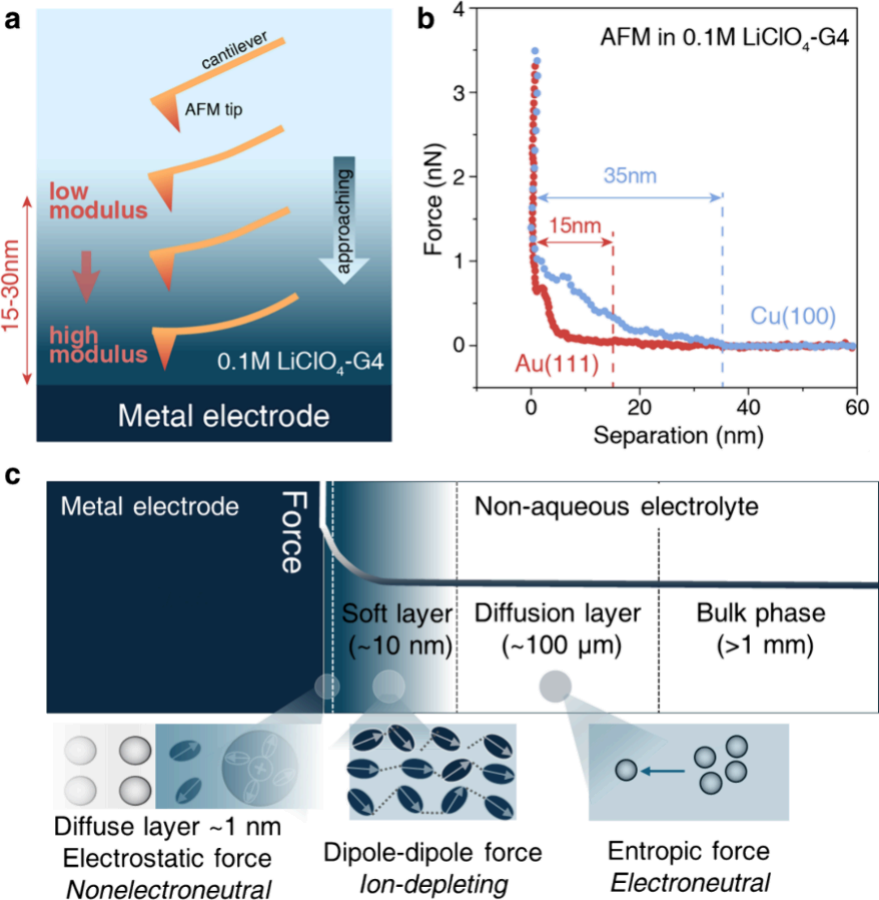
Soft layer at the metal–nonaqueous solution interfaces with an ultralow modulus. (a) Schematics of the AFM-based force spectroscopy at the metal–nonaqueous solution interface. (b) AFM force curves obtained at Au(111) and Cu(100) in 0.1 M LiClO_4_-G4 confirm the presence of a soft interfacial layer with a low modulus of 2 MPa. (e) Schematics of the metal–nonaqueous solution interface featuring the newly observed soft layer, sandwiched between the diffuse layer and the diffusion layer.

Atomic force microscopy (AFM)-based force spectroscopy is a powerful method to obtain structural information at the electrochemical interfaces.^40,41^ AFM operates using a sharp probe, typically fabricated from silicon or silicon nitride mounted on a flexible cantilever. The tip of the probe interacts with the sample surface through interatomic forces. As the tip scans the surface in close proximity, the interactions induce a deflection of the cantilever. A laser beam is directed onto the back of the cantilever and reflected onto a position-sensitive detector. Deflection or oscillation of the cantilever leads to a corresponding shift in the position of the reflected laser spot on the position-sensitive detector. The resulting displacement signal provides a highly accurate measurement of the interaction forces between the probe and the sample surface, enabling atomic scale/nanoscale characterization of mechanical properties. Herein, we conduct AFM-based force curve measurements to probe this interfacial layer (see Figure 2a). A nonvolatile 0.1 M LiClO_4_-tetraethylene glycol dimethyl ether (G4) electrolyte was used to replace the 0.1 M LiClO_4_-DME electrolyte. Force curves at Au, Cu, and highly oriented pyrolytic graphite (HOPG) electrodes were recorded in 0.1 M LiClO_4_-G4 in an Ar-filled glovebox. The experimental details are described in Materials and Methods. The AFM force curve obtained at the Au/LiClO_4_-G4 interface is shown in Figure 2b. It shows an interfacial layer at around 15 nm. Similarly, an interfacial layer of around 35 nm can be also observed at the Cu/LiClO_4_-G4 interface, as shown in Figure 2b, which is thicker than that of the Au/LiClO_4_-G4 interface. However, the AFM force curve at the HOPG/LiClO_4_-G4 interface just shows a sharp slope without steps (Figure S7), suggesting that the interfacial layer could not be identified at HOPG.

To understand the results from AFM force curves, EIS measurements were conducted at Au, Cu, and HOPG electrodes in 0.1 M LiClO_4_-G4 (Figures S8 and S9). *L* at these electrodes was calculated and is shown in Table S1. We find that the thicknesses of the interfacial layers at the Au and Cu electrodes determined by EIS are around tens of nanometers and that the interfacial layer at the Cu electrode is indeed thicker than that at the Au electrode. It is worth noting that the estimated *L* at HOPG from EIS is 154 nm, which is much thicker than those at Au and Cu electrodes. The much thicker interfacial layer could be too soft to be detected for AFM. Therefore, the above data from EIS measurements can still be reconciled with observations by AFM force curves. Since the Au and Cu electrodes used herein had not been reduced to potentials negative of 2 V (vs Li^+^/Li), the electrolyte decomposition and consequent SEI formation were avoided, which excludes the possibility that this interfacial layer is the SEI layer. Furthermore, for both Au and Cu electrodes, the Young’s moduli of the interfacial layers are about 2 MPa, which is nearly 2 orders of magnitude lower than that of organic-rich SEI formed at lithium anodes (typically several hundreds of MPa), demonstrating that softness is a feature of the interfacial layer.^42^

In summary, the high-frequency capacitance is estimated to be only 0.38 μF cm^–2^ for Au in 0.1 M LiClO_4_-DME solution, which is 2 orders of magnitude smaller than the normal values of *C*_dl_. This ultralow capacitance is attributed to the geometric capacitance of an ionically conductive layer near the metal surface. In addition, the ionic conductivity of this layer is 5 orders of magnitude lower than the conductivity of bulk electrolytes (0.78 mS cm^–1^) and it is even several orders lower than the polymer-based solid state electrolyte (i.e., 0.01–0.1 mS cm^–1^). AFM unambiguously confirms that there is indeed an electrolyte layer of several tens of nanometers more rigid than the bulk electrolyte solution but much softer than a SEI layer. Combined, we term this layer as a soft layer. A schematic diagram of the metal–solution interface including the soft layer is shown in Figure 2c. Due to the difference in thickness, the soft layer (10–100 nm) is a layer beyond the double layer (∼1 nm). In what follows, we systematically study how this soft layer changes with the electrode material, electrolyte solution, and temperature.

### Influencing Factors of the Soft Layer

**Figure 3:**
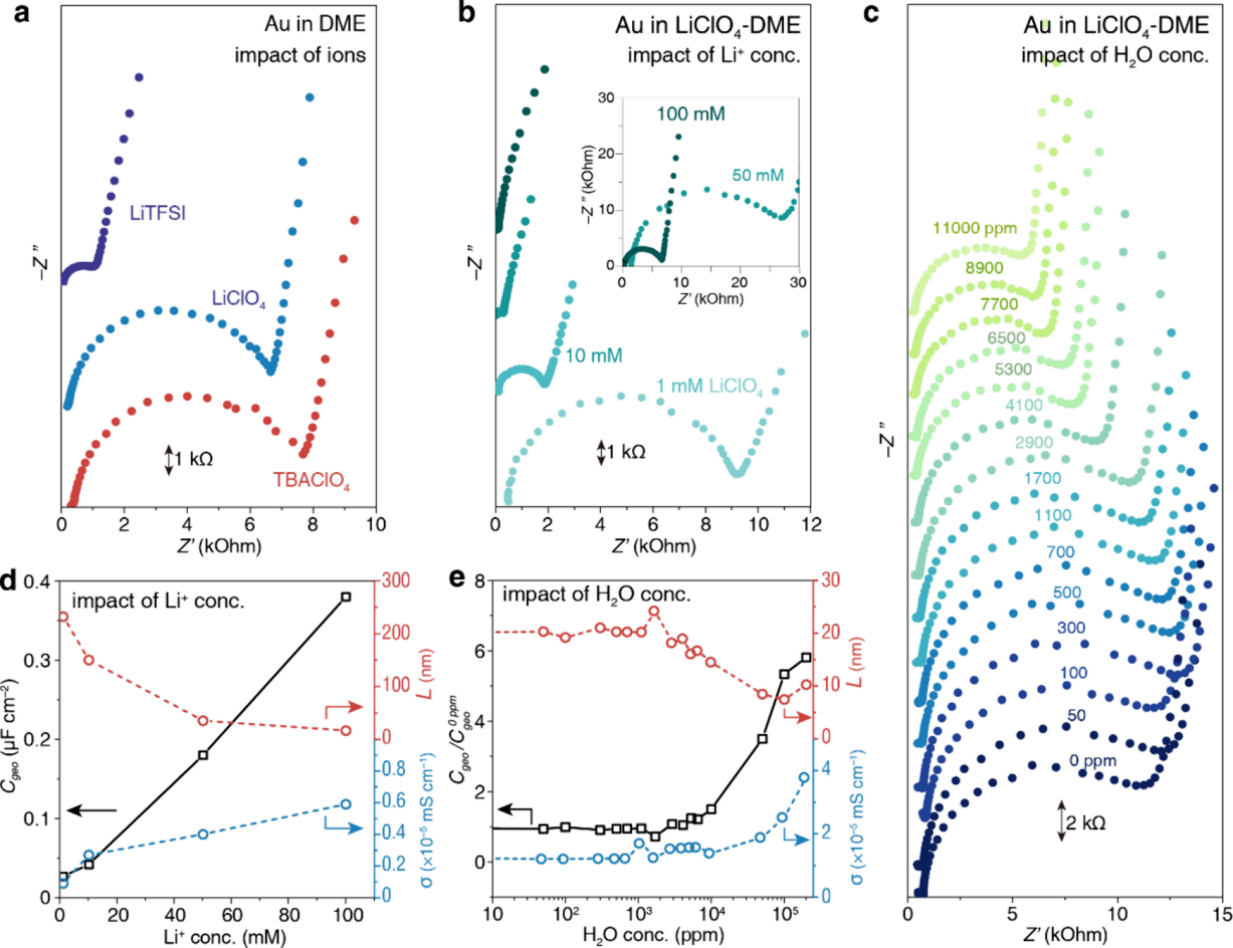
Impact of cations, anions, salt concentrations, and water concentrations on the EIS plots and properties of the soft layer. (a–c) Nyquist plots of the EIS in electrolytes containing (a) 0.1 M LiClO_4_, 0.1 M TBAClO_4_, and 0.1 M LiTFSI; (b) various concentrations of LiClO_4_ and (c) various water concentrations in the electrolyte at the Au electrode. The plots were shifted vertically for clarity, and the scale bars of the *y*-axis are marked in (a–c). (d, e) Trend of the capacitance (*C*_geo_), layer thickness (*L*), and AC ionic conductivity (σ) in the soft layer with different (d) salt concentrations and (e) water concentrations.

To further explore the properties of the soft layer, we systematically examine the following factors, including the type and concentration of ions, the water content in electrolytes, and the temperature. First, the impact of cations and anions is studied. As shown in Figure 3a, *C*_geo_ values for TBAClO_4_ and LiClO_4_ are very close, viz., 0.35 and 0.38 μF cm^–2^ (Table 1), respectively. In contrast, the anions exhibit a greater effect on the semicircle, as shown in Figure 2b. The *C*_geo_ in LiTFSI-DME is 1.48 μF cm^–2^, which is about 4-fold higher than the *C*_geo_ in LiClO_4_-DME, implying that the thickness of the soft layer in LiClO_4_ is 4-fold thicker than that in the LiTFSI electrolyte.

**Table 1:** Physical Properties of the Soft Layer at Au and Cu Electrodes in Ether-Based Electrolytes Obtained Using a Physical-Based EIS Model^a^

**electrode/solution**	**salt**	***C***_**geo**_ (μF cm^–2^)	***L*** (nm)	**σ** (×10^–5^ mS cm^–1^)
Au/DME	100 mM LiTFSI	1.48	4.30	1.14
	100 mM TBAClO_4_	0.35	18.40	0.78
	100 mM LiClO_4_	0.38	16.64	0.59
	50 mM LiClO_4_	0.18	35.25	0.40
	10 mM LiClO_4_	0.04	150.47	0.27
	1 mM LiClO_4_	0.03	232.35	0.09
Cu/DME	100 mM LiTFSI	0.60	10.84	2.77
	100 mM TBAClO_4_	0.15	43.54	2.28
	100 mM LiClO_4_	0.18	35.44	1.28
	50 mM LiClO_4_	0.10	66.85	0.87
	10 mM LiClO_4_	0.039	166.45	0.41
	1 mM LiClO_4_	0.027	234.39	0.10
Au/G4	100 mM LiClO_4_	0.38	17.73	0.63
	50 mM LiClO_4_	0.11	51.8	0.63
	10 mM LiClO_4_	0.081	83.9	0.24
	1 mM LiClO_4_	0.048	140.6	0.08
Cu/G4	100 mM LiClO_4_	0.19	36.58	1.35
	50 mM LiClO_4_	0.10	67.67	0.92
	10 mM LiClO_4_	0.078	87.78	0.24
	1 mM LiClO_4_	0.031	217.53	0.11

a *C*_geo_, *L*, and σ: geometric capacitance, thickness, and AC conductivity of the soft layer.

Second, the concentration of electrolyte salt influences markedly the semicircles in EIS and *C*_geo_, as shown in Figure 3c,d. Here, ϵ changes barely in the examined range of ionic concentration.^43^ With the increase in salt concentration, *L* decreases from about 200 nm at 1 mM to around 20 nm at 100 mM. Along with thinning, σ increases from 0.9 to 5.9 nS cm^–1^.

Third, recognizing the significant role of water in the soft layer, we conducted EIS measurements in a series of DME–water mixture electrolytes. Illustrated in Figure 3e and Figure S10, the semicircle in Nyquist plots gradually decreases, while *C*_geo_ and σ steadily increase with the rising water concentration in electrolytes (Figure 3e). Simultaneously, the thickness of the soft layer decreases at a higher water content. Following this trend, the soft layer completely disappears in aqueous solution (see Figure S2).

**Figure 4:**
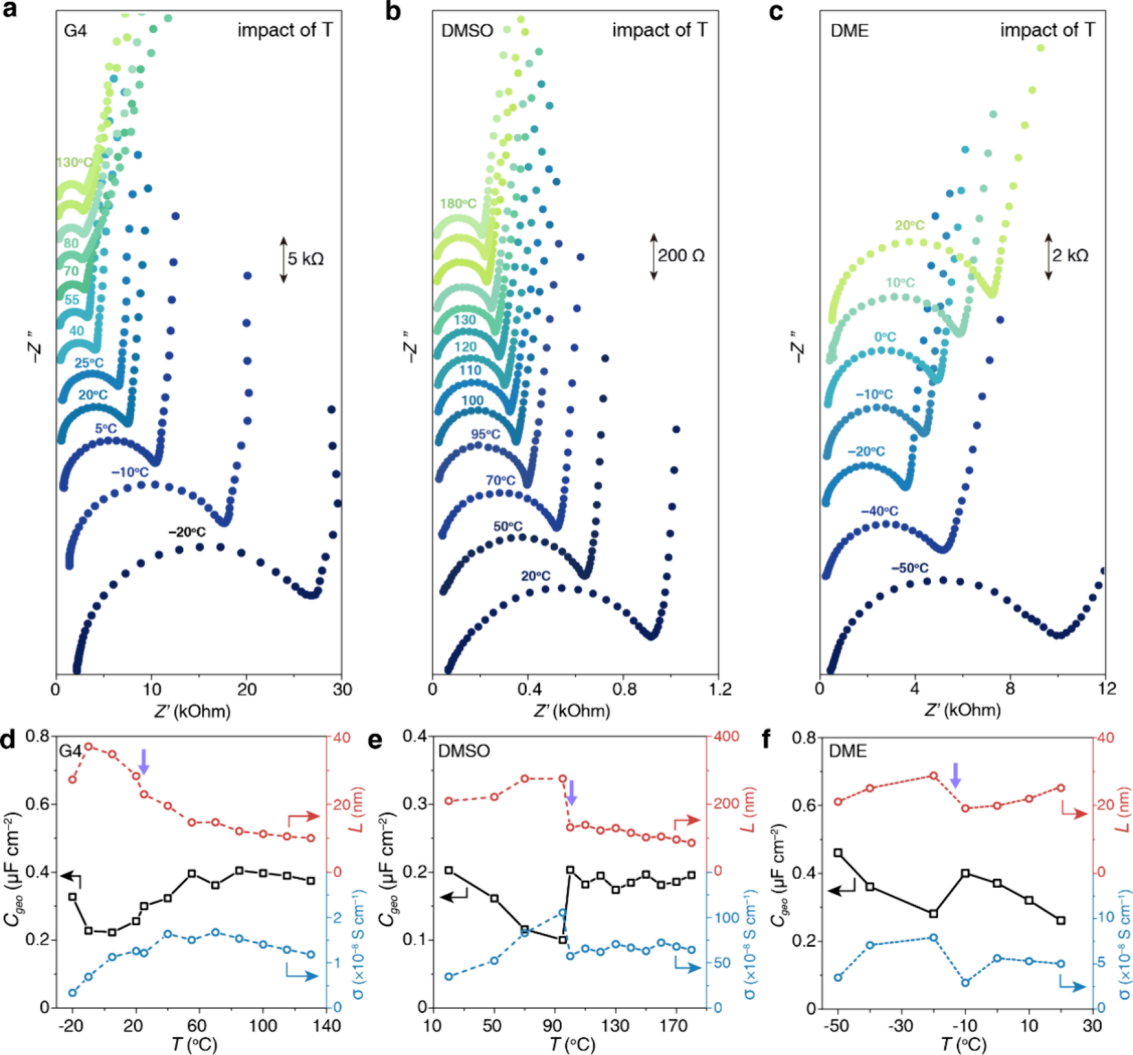
Impact of temperature on the soft layer. (a–c) Nyquist plots of the EIS at the Au electrode (a) in 0.1 M LiTFSI-G4, (b) in 0.1 M LiTFSI-DMSO, and (c) in 0.1 M LiClO_4_-DME at various temperatures. The plots were shifted vertically for clarity and the scale bars of the *y*-axis are marked in (a–c). (d–f) Corresponding thickness and AC ionic conductivity of the soft layer as a function of temperature. EIS was recorded in three-electrode cells from 1 MHz to 0.1 Hz. Extraction of the physical properties of the soft layer from the EIS data is detailed in the Materials and Methods Section.

Fourth, the temperature dependence of the soft layer was studied. EIS experiments were conducted on Au in 0.1 M LiTFSI-G4, 0.1 M LiTFSI-DMSO, and 0.1 M LiClO_4_-DME at various temperatures, as illustrated in Figure 4. Because LiClO_4_ is explosive when heated with organics, LiTFSI was used in G4 and DMSO electrolyte for high-temperature measurements. Overall speaking, the soft layers with DMSO and DME become thinner and more ionically conductive at higher temperatures. Most interestingly, we observe a sharp decrease in the thickness of the soft layer at 95 °C for DMSO, as shown in Figure 4b, which implies a sudden breakdown of the layer due to the increased thermal forces. This temperature is equivalent to an intermolecular force of ∼3 kJ/mol, falling within the region of dispersion forces, namely, the fluctuating dipole–induced dipole interaction.^44,45^ Similar behaviors are also observed for G4 and DME but at much lower temperatures, implying weaker dispersion forces. This trend is also consistent with the fact that the dipole moment of DMSO is the largest among the three solvents. We observe a reformation of the soft layer after its breakdown. As the glass cell was heated up from 95 to 100 °C and then cooled back to 95 °C within 10 min, the two curves at 95 °C overlaps, suggesting a quick reformation of the soft layer (Figure S11). A detailed study of the temperature-induced deformation of the soft layer is beyond the scope of this work.

## Conclusions

Our impedance measurements at various metal–nonaqueous solution interfaces exhibit a high-frequency semicircle prevalent across a wide range of ideally polarizable conditions. With the aid of a physical impedance model, the capacitance and resistance associated with this high-frequency semicircle, denoted *C*_hf_ and *R*_hf_, respectively, have been extracted. *C*_hf_ is 1–3 orders of magnitude lower than the double layer capacitance. Substantiated by the AFM results, *C*_hf_ represents the geometry capacitance of a quasi-solid, electrolytic, soft layer of 10–100 nm.

The thickness and AC ionic conductivity of this soft layer were studied as a function of the electrode substrate, solvent, ions, salt concentrations, water concentrations, and temperature. First, the properties of this soft layer are largely independent of the electrode potential. However, they are sensitive to the electrode substrate and the solvent. Interestingly enough, its thickness decreases with increasing water content, and it completely disappears in aqueous solutions. Second, the soft layer is strongly dependent on the ion concentration. Its thickness decreases at higher ion concentrations. Interestingly, it is more sensitive to the anion identity than to the cation identity. Its AC ionic conductivity is even several orders lower than that of polymer-based electrolytes. Third, the soft layer becomes thinner at higher temperatures, with a sharp drop in thickness at a critical temperature.

The above experimental clues lead us to speculate that this soft layer originates from metal-mediated weak long-range intermolecular forces among solvent molecules. It is important to note that the order in the first few layers of solvent molecules, thanks to short-range interactions with the metal surface, is central to the formation of the soft layer. Recent *ab initio* molecular dynamic (AIMD) simulations have revealed that water molecules are chemisorbed on transition metals to a varying extent.^46,47^ Therefore, with a trace amount of water in organic solvent, the order in the first few layers of organic solvent is disturbed by the chemisorbed water molecules, resulting in a thinner soft layer or even the absence of the soft layer. The same reasoning applies to interpreting why anions have a larger influence on the soft layer than cations, considering that anions are more easily specifically adsorbed on the metal surface. Since this soft layer does not disappear at elevated temperatures in the examined solvents, we conjecture that its formation is dominated by enthalpic effects rather than entropic effects. Moreover, the ion concentration dependence suggests that the enthalpic effects are dramatically weakened at higher concentrations of ions. Combined, the multifaceted analysis leads us to conclude that weak, long-range intermolecular forces are mediated mainly via dipole–dipole interactions. We note that the dipole moment might be an oversimplified descriptor of the solvent effect on the soft layer, calling for more detailed studies of the influence of the atomic properties of the solvent in the future. The low conductivity is due to the low mobility and/or low concentration of the ions in the soft layer. As the soft layer is quasi-solid, the activation energy of ion hopping is likely to be higher than that in liquid electrolytes. In addition, the formation of ion pairs in the soft layer could also decrease the concentration of free ions. Together, both factors lead to the observed much lower conductivity.

This work shows that the interfacial regions between the electrode and nonaqueous electrolytes are different from the counterparts in aqueous electrolytes. The insights may help understand the electrochemical behavior in the systems using nonaqueous electrolytes, like metal deposition and SEI formation in Li/Na-ion batteries, organic electrocatalysis and synthesis, *etc*. Specifically, this soft layer is detrimental to the dynamics and kinetics of metal–nonaqueous solution interfaces for two reasons. On the one hand, the AC ionic conductivity is much lower in this soft layer than in the bulk solution. On the other hand, the time constant of charging this soft layer is much larger than that of charging the EDL. Our study shows that this soft layer can be thinned effectively by increasing the ion concentration and mixing the organic solvent with a trace amount of water. This phenomenon may be a common occurrence at the metal–organic solution interface, not just limited to the electrode surface. Observation of this nanoscale soft layer opens new avenues of tuning the local reaction conditions for SEI formation and ion transport properties, which are crucial to battery performance. We hope a more detailed mechanistic picture of the soft layer will be unraveled in the near future with the aids of the emerging machine-learning force field-based molecular simulations.

## Data Availability

All data needed to evaluate the conclusions are present in the paper and/or Supporting Information.

## Supporting Information Available

Details of experiments, including electrochemical measurements, NMR measurements, roughening of the gold electrode, AFM measurements, and physical model analysis (PDF)
