# Supplementary material for: Structural Basis of Ultralow Capacitances at Metal–Nonaqueous Solution Interfaces
Source: J Am Chem Soc. 2025 Jan 27;147(5):4060–8. doi: 10.1021/jacs.4c12443 (PMC11803618; doi:10.1021/jacs.4c12443)
Supplement: Supplementary file 1 — ja4c12443_si_001.pdf [file ja4c12443_si_001.pdf]

Supporting Information for

Structural Basis of Ultralow Capacitances at Metal–Nonaqueous Solution Interfaces

Juan Chen[1, ‡], Zengming Zhang[2, ‡], Xiaoting Yin[3, ‡], Chenkun Li[2], Fengjiao Yu[1], Yuping Wu[1,4], Jiawei Yan[3,*], Jun Huang[2,5,*], Yuhui Chen[1,*]

[1]State Key Laboratory of Materials-Oriented Chemical Engineering, College of Chemical Engineering, Nanjing Tech University, Nanjing 211816, China

[2] Institute of Energy Technologies, IET-3: Theory and Computation of Energy Materials, Forschungszentrum Jülich GmbH, 52425 Jülich, Germany

[3] State Key Laboratory of Physical Chemistry of Solid Surfaces and College of Chemistry and Chemical Engineering, Xiamen University, Xiamen 361005, China

[4] Key Laboratory of Energy Thermal Conversion and Control of Ministry of Education, School of Energy and Environment, Southeast University, Nanjing 210096, P. R. China.

[5] Theory of Electrocatalytic Interfaces, Faculty of Georesources and Materials Engineering, RWTH Aachen University, Aachen 52062, Germany

[‡] These authors contributed equally to this work.

Materials and Methods

Electrochemical measurements

A three-electrodes cell was applied for the EIS measurement. In most experiments, a planar gold electrode (2 mm diam.) served as the working electrode (WE), and a platinum (Pt) electrode served as the counter electrode (CE). A nonaqueous Ag reference electrode in a glass tube with a glass frit at the end was employed. In aqueous electrolyte like 0.1M $LiClO_4$-water, a commercial AgCl/Ag reference electrode was used. The Au working electrode was polished with the polishing liquid (Al_2O_3, CHI Inc) first and then we ran cyclic voltammetry in 0.5 M H_2SO_4 solution to obtain a clean surface. To study the impact of electrode substrate, various electrodes such as Pd disk (2 mm diam.), Pt disk (2 mm diam.), and Ni wire (0.5 mm diam.) were used. Surface area of Ni electrode is 0.47 cm^2, the surface area of Au, Pd, and Pt electrodes are 0.031 cm^2. To prevent the Ni from oxidation at ambient atmosphere, the Ni wire was polished using sand paper inside the Ar-filled glovebox just before EIS measurements.

Dimethyl sulfoxide (DMSO), tetraethylene glycol dimethyl ether (G4), acetonitrile, and 1,2-dimethoxyethane (DME) were purchased from Macklin. All solvents must be dried over molecular sieves and distilled under Ar (or vacuum) before use. The water content of the whole electrolyte was tested by Karl Fischer Moisture Titrator and was less than 1 ppm. The tetra-butylammonium perchlorate and lithium perchlorate (Aldrich) were recrystallized and dried at 100^oC under vacuum prior to use. LiTFSI (Aldrich) was dried at 120^oC under vacuum prior to use.

EIS measurements were carried out in an Ar-filled glovebox (O_2 < 0.1 ppm, H_2O < 0.1 ppm) using a CHI660E potentiostat. The frequency range was 1 MHz~1 Hz and the amplitude of the potential signal was 10mV. For EIS with different amount of water, the electrolyte with different volume concentration of water was prepared.

For EIS at low temperatures, the 3-electrode cell was placed in a cryostat. For high temperatures, the cell was connected to a distillation setup with Ar purging and heated in an oil bath. EIS was tested after the temperature became stable.

^1H NMR

The electrolyte used in the experiment was added to D_2O for ^1H NMR (Bruker 400MHz) to rule out the impurities in the electrolyte.

Gold electrode roughening

To obtain a roughened gold, we followed the oxidation and reduction cycle(s) (ORC) procedure developed by Weaver and coworkers. The Au electrode was first immersed in 0.1M H_2SO_4 solution to conduct cleaning electrochemically in the potential range of-0.25 and 1.5 V. After rinsing with ultrapure water, the Au electrode was kept at -0.3 V in 0.1M KCl until the current was stable. Then, the potential was scanned to 1.2 V at 1V/s and set for 1.2 s for oxidation, after then scanned back to -0.3 V at 0.5 V/s and set for 30 s for reduction. The potential cycle was repeated for about 15 min and the final potential should be -0.3 V to ensure a reduced state of the electrode. This roughening process resulted in an Au surface with a brown appearance. The rough Au was used as WE in 0.1M $LiClO_4$ DME to conduct PEIS test. The result was shown in Figure S4.

AFM Measurement

All AFM (Bruker Dimensional Icon) characterizations were conducted in an Ar-filled glovebox (MIKROUNA, H_2O < 0.1 ppm, O_2 < 0.1 ppm). Force curves of the sample were obtained by the force spectroscopy mode by using a CSG10 probe (TipsNano) with a spring constant of 0.11 N m^{-1} at the open circuit voltage. Au(111), Cu(100), and freshly cleaved HOPG were respectively used as

working electrodes.

Model fitting

In this part, we explain how we obtained the R_{hf} and C_{hf} from experimental data using the physical model. In the high-frequency region, this physical model can be transformed into an RC electric circuit, illustrated in Figure S12a. We fit the model with the experimental data to obtain R_{hf} and C_{hf}. The fitting results of the EIS depicted in Figure. 1 are presented in Figure S12b.

Solution permittivity ϵ

The bulk permittivities of the electrolyte solutions at room temperature are

	ACN	DMSO	DME	G4
ϵ/ϵ_0	37.40	46.80	7.20	7.68

The temperature dependence of the permittivity is considered in the analysis of temperature-varying EIS results according to [2],

$$\log \epsilon_{\mathrm{eff}}(T) = \log(7.2) - 2.5 \times 10^{-3}(T * \mathrm{K}^{-1} - 298) \quad \text{(S1)}$$

For mixtures of water and nonaqueous solvents, we use a linear relation to estimate the permittivity [2],

$$\epsilon_{\mathrm{eff}}(C_{water}) = 7.2 + 0.713 \cdot C_{water} \quad \text{(S2)}$$

where the C_{water} is the water content expressed as a percentage.

Supplementary figures

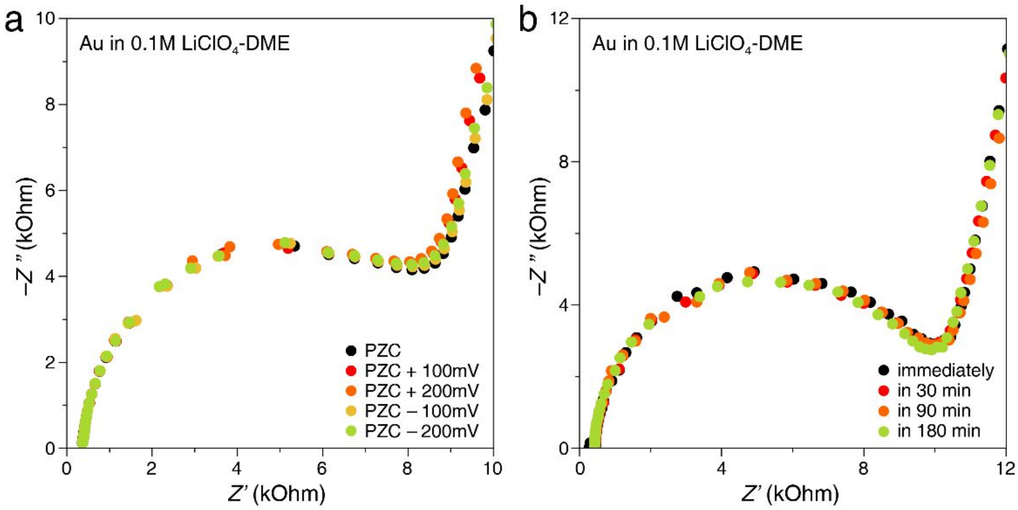

Figure S1. Impact of overpotential and rest times on the EIS plots. Nyquist plots of the EIS with (a) different overpotentials from -200 mV to 200 mV vs PZC and (b) various periods of rest time after being immersing into the electrolyte. EIS was recorded at an Au electrode in 0.1 M $LiClO_4$-DME in three-electrode cells from 1 MHz to 0.1 Hz. The identical semicircles suggest that it is not from R_{ct} and it is independent of the immersion time.

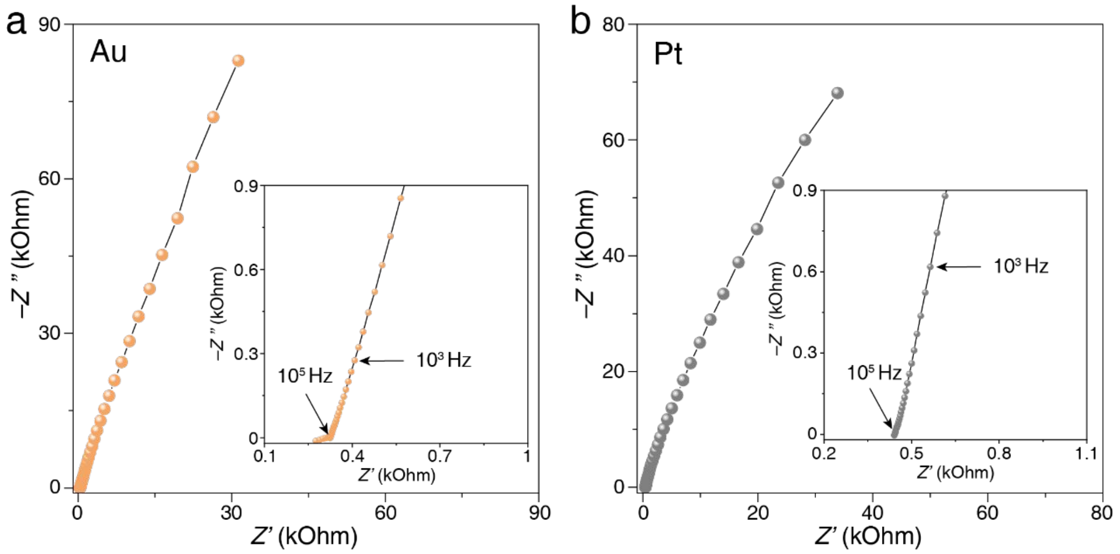

Figure S2. Nyquist plots of the EIS at (a) Au and (b) Pt electrodes in 0.1 M $LiClO_4$-H_2O electrolyte. EIS was recorded in three-electrode cells from 1 MHz to 1 Hz. No semicircle was identified at the high Frequency region.

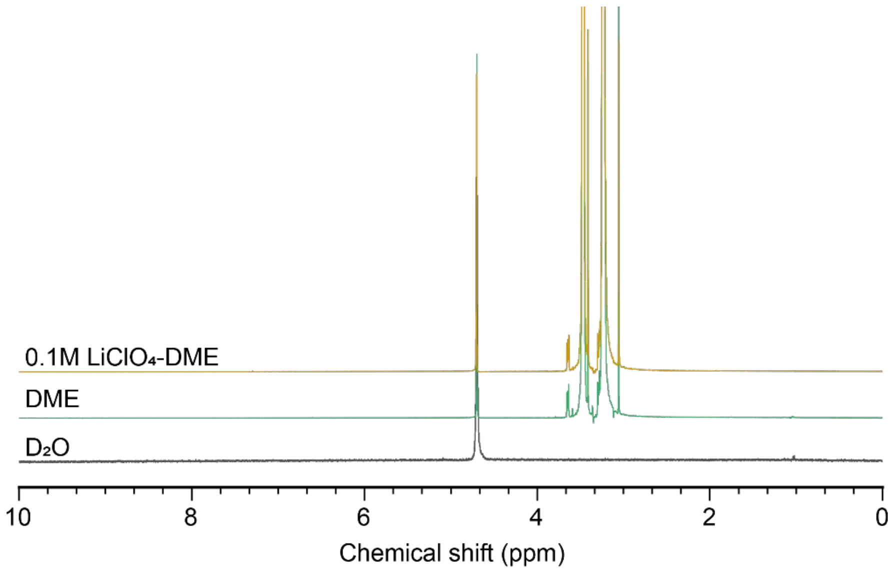

Figure S3. ^{1}H NMR spectrum of the DME-based electrolyte.

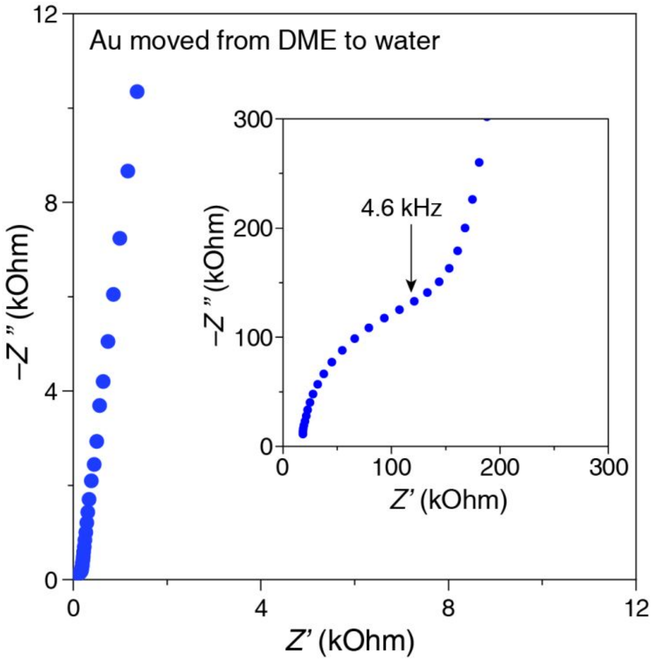

Figure S4. Nyquist plots of the EIS at Au electrode move from 0.1 M $LiClO_4$-DME to 0.1 M $LiClO_4$-H_2O. This semicircle disappears with time and this state is not a stable and steady state.

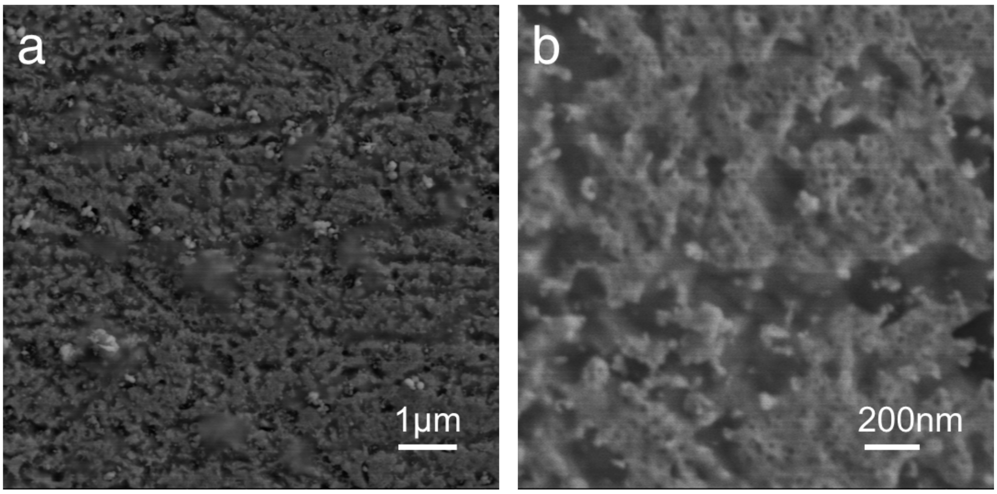

Figure S5. SEM images of the Au electrode that has been electrochemically roughened in 0.1 M KCl-H_2O.

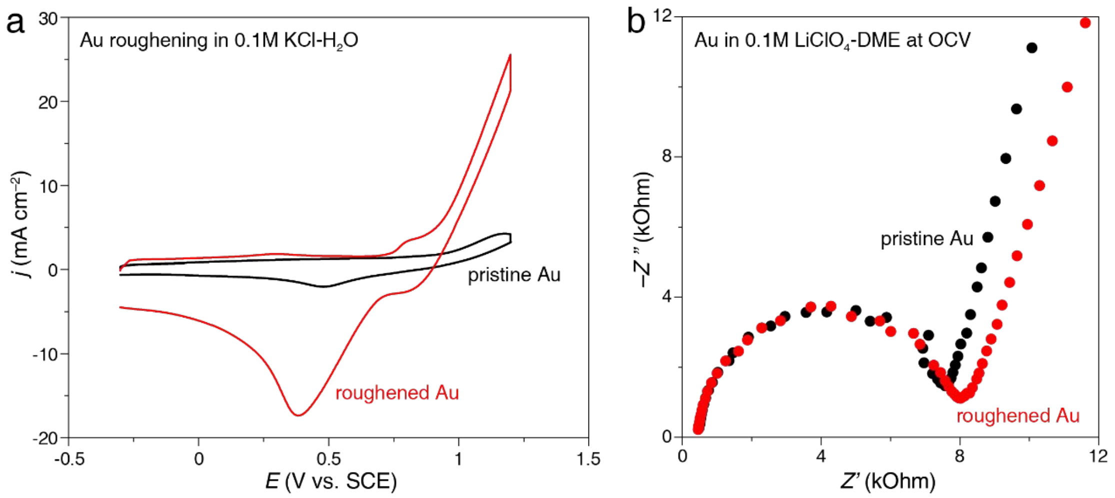

Figure S6. (a) CV of the Au electrode before and after roughening in KCl-H_2O solution. (b) Nyquist plots of the EIS at pristine Au and roughened Au electrodes in 0.1 M $LiClO_4$-DME.

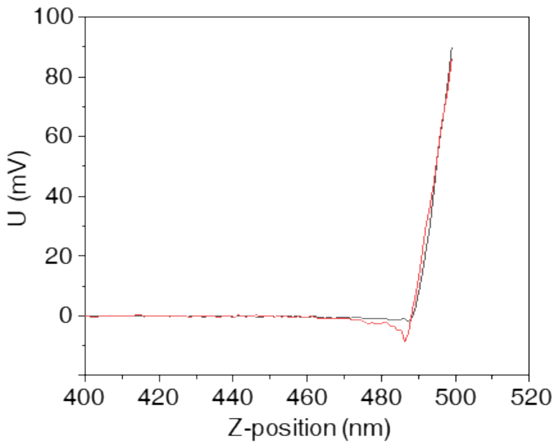

Figure S7. AFM peak force curve at HOPG surface in 0.1 M $LiClO_4$-G4 at the PZC.

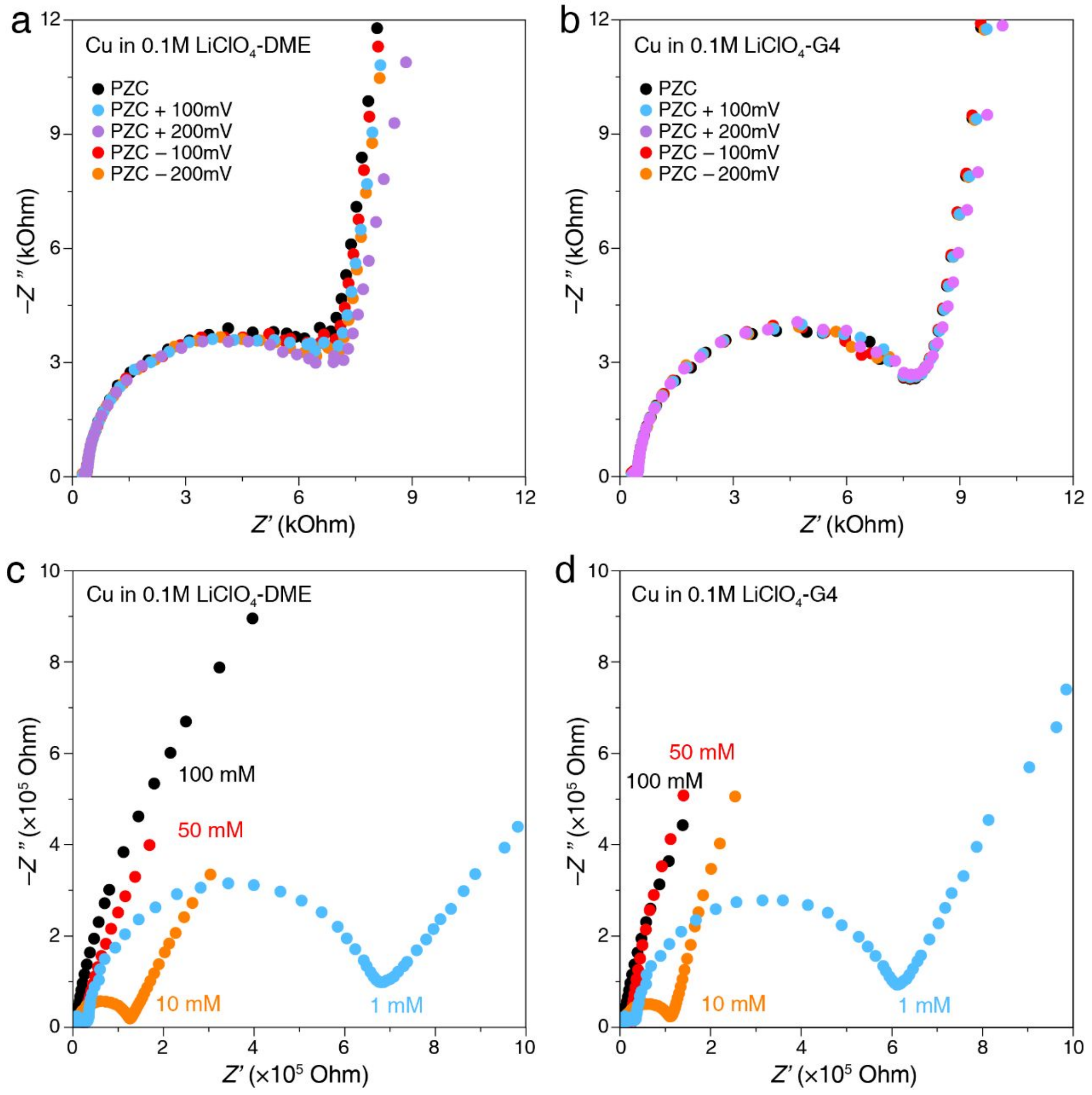

Figure S8. Nyquist plots of the EIS at Cu electrode (a,b) with different overpotentials from -200 mV to 200 mV vs PZC in (a) 0.1 M $LiClO_4$-DME and (b) 0.1 M $LiClO_4$-G4. EIS at PZC with various concentration of $LiClO_4$ at Cu electrode in (c) DME and (d) G4.

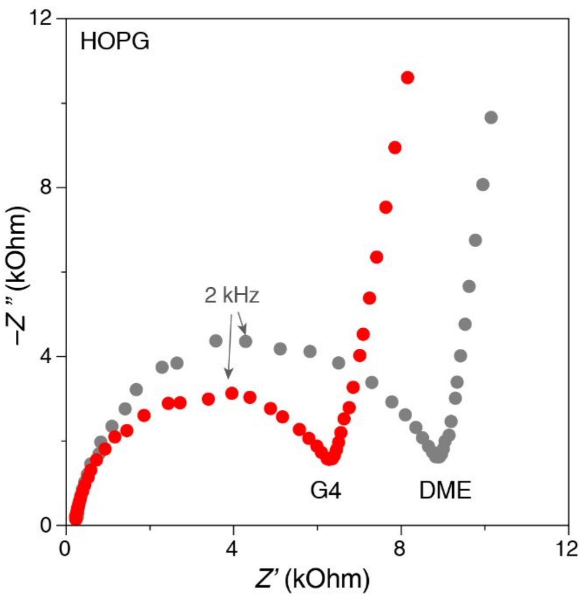

Figure S9. Nyquist plots of the EIS at a HOPG electrode in 0.1 M $LiClO_4$-DME and 0.1 M $LiClO_4$-G4.

Table S1. Thickness of the soft layer at various electrodes in DME and G4.

L (nm)	**Au**	**Cu**	**HOPG**
**0.1M $LiClO_4$-DME**	16.64	35.44	100.71
**0.1M $LiClO_4$-G4**	17.73	36.58	154.12

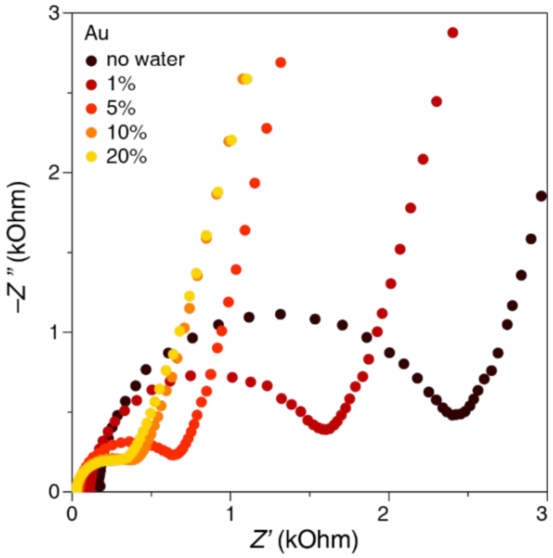

Figure S10. Nyquist plots of the EIS at Au electrode in 0.1 M $LiClO_4$-DME without water and with 1%, 5%, 10%, and 20% of water. EIS was recorded at PZC in three-electrode cells from 1 MHz to 0.1 Hz. The electrode area is 0.0707 cm^2.

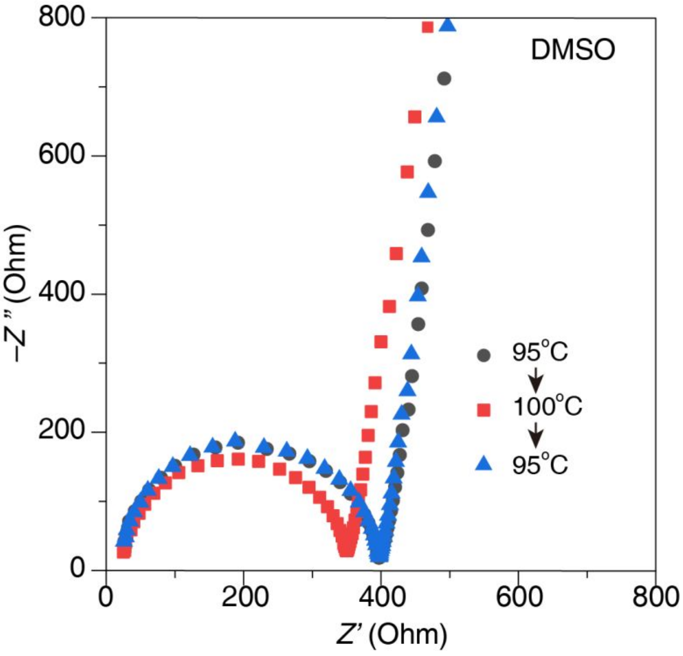

Figure S11. Nyquist plots of the EIS at Au electrode in 0.1 M LiTFSI-DMSO. The glass cell was heated up from 95°C to 100°C then cooled back to 95°C. The overlapping curves at 95°C suggests the reformation of the soft layer.

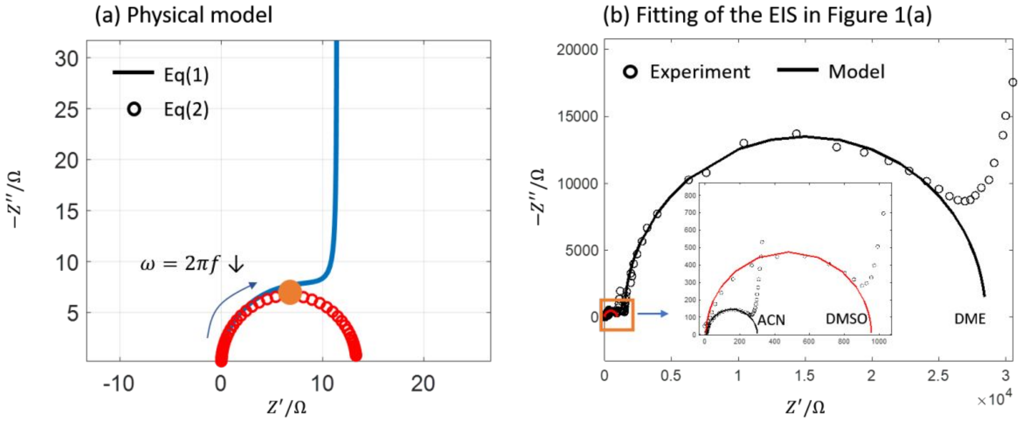

Figure S12. (a) Nyquist plots of the physical model in Eq. (1) and Eq. (2). (b)Fitting of the EIS in Figure. 1(a).

Supplementary references

1. Gao, P.; Gosztola, D.; Weaver, M. L., Coupling real-time surface-enhanced raman spectroscopy with linear-sweep voltammetry: Application to elucidation of electrochemical mechanisms. *Anal. Chim. Acta* **1988,** *212*, 201-212.

2. Yao, N.; Chen, X.; Shen, X.; Zhang, R.; Fu, Z.-H.; Ma, X.-X.; Zhang, X.-Q.; Li, B.-Q.; Zhang, Q., An Atomic Insight into the Chemical Origin and Variation of the Dielectric Constant in Liquid Electrolytes. *Angew. Chem. Int. Ed.* **2021,** *60* (39), 21473-21478.
